# Co-expression of estrogen receptor-alpha and targets of estrogen receptor action in proliferating monkey mammary epithelial cells

**DOI:** 10.1186/bcr1374

**Published:** 2006-01-03

**Authors:** Constantine Dimitrakakis, Jian Zhou, Jie Wang, Ludmila Matyakhina, Eva Mezey, Jesse Xiyu Wood, Daniel Wang, Carolyn Bondy

**Affiliations:** 1 Developmental Endocrinology Branch, NICHD, NIH, Bethesda, MD 20892, USA; 2 NIDCR, NIH, Bethesda, MD 20892, USA

## Abstract

**Introduction:**

Failure to detect co-expression of estrogen receptor-alpha (ERα) and proliferation 'markers' such as Ki67 in human mammary epithelium led to the view that estrogen acts indirectly to stimulate mammary epithelial proliferation. The mitotic index was so low in prior studies, however, that transient co-expression of ERα and Ki67 during the cell cycle could have been below detection limits.

**Methods:**

Immunohistochemistry was used on mammary tissue sections from estrogen treated rhesus monkeys to investigate co-expression of ERα and the proliferation antigen Ki67. Using the same methods, we investigated the cell localization of proteins involved in estrogen-induced proliferation, including cyclin D1, stromal cell-derived factor (SDF)-1, and MYC.

**Results:**

ERα was co-expressed with the proliferation marker Ki67 as well as with SDF-1, MYC and cyclin D1 in mammary epithelial cells from estrogen-treated monkeys.

**Conclusion:**

ERα is expressed in proliferating mammary epithelial cells together with the estrogen-induced proteins MYC, cyclin D1 and SDF-1, consistent with a direct mitogenic action by estrogen in primate mammary epithelium.

## Introduction

Estrogen receptors (ERα and ERβ) belong to the nuclear receptor family and function as hormone inducible transcription factors in target cells [1]. ERα is thought to mediate the mitogenic actions of estrogen by inducing the expression of genes involved in cell proliferation, including cyclin D1, MYC and stromal cell-derived factor (SDF)-1 [2,3]. Failure to detect co-expression of ERα and Ki67 (a proliferation marker) in normal human mammary epithelium led to the proposal that estradiol acts indirectly in this tissue by stimulating ER-positive epithelial cells to release (unknown) mitogens that then trigger proliferation in neighboring ER-negative epithelium [4,5]. Of note, the mammary epithelial proliferation index in these studies was very low, generally less than 5% and often closer to 1%.

Estrogen stimulates breast cancer cells in G0 to enter G1 and progress to S phase [6], and thus one might expect to find ERα expression in some G0 cells and through some part of G1 phase in proliferating cells. As with most cell cycle regulatory proteins, Ki67 expression is tightly regulated, appearing in mid G1 with a half-life of 60 to 90 minutes [7]. Thus, it appears likely that during the normal mitotic cycle, ERα and Ki67 may be co-expressed for a limited period beginning in G1. The number of cells in G1 and the short duration of co-expression result in a low probability of detecting the transient co-expression.

To improve our understanding of ERα action in primate mammary epithelium, in the current study we have investigated the co-expression of ERα, the proliferation marker Ki67 and estrogen target proteins MYC, cyclin D1 and SDF-1 in the highly proliferative mammary epithelium from estrogen-treated rhesus monkeys.

## Materials and methods

We have previously shown that ovariectomy followed by short term estrogen treatment greatly increases the mammary epithelial proliferation index in the rhesus monkey [8]. We thus investigated ERα and Ki67 expression using immunohistochemical detection on thin (3 μm) serial sections through the mammary epithelium in estrogen-treated monkeys. Details of the treatment and immunohistochemical protocols have been published [8].

For double immunofluorescence of ERα, MYC and SDF-1, frozen sections (5 μm) of monkey mammary gland biopsies were fixed in cold methanol for 4 minutes, washed twice in PBS and then incubated with 8% bovine serum albumin for 1 h at room temperature. The following antisera were used: ERα and Ki-67 mouse monoclonal (Novocastra Laboratories Ltd, Newcastle upon Tyne UK), SDF-1-α rabbit polyclonal (Leinco Technologies Inc., St. Louis, MO, USA) and C-MYC mouse monoclonal (NeoMarkers, Fremont, CA, USA). The dilution 1:50 was used for primary antibodies. Secondary antibodies, anti-rabbit Ig-FITC and anti-mouse Ig-Cy3 were from Sigma (Sigma-Aldrich, St. Louis, MO USA) and Caltag Laboratories (Burlingame, CA USA), respectively. The slides were stained with 4',6-diamidino-2-phenylindole (DAPI) and examined using a Leica epifluorescence microscope; fluorescence images were automatically captured on a Photometrics cooled-CCD camera (Photometrics Ltd, Tuscon, AZ, USA), using IP Lab Image software (Scanalytics Inc., Fairfax, VA, USA).

For double immunofluorescence of ERα and cyclin D1, the tissues were fixed in 8% buffered paraformaldehyde for 10 minutes at room temperature. Following rinses in PBS, the slides went through heat induced antigen retrieval. The non-specific sites were blocked with 1 × Power-block (Zymed, South San Francisco, California, USA) and then incubated in the ERα antibody (Novocastra F611) at a 1:200 dilution overnight at 4°C. Biotinylated anti-mouse (1:200) and ABC (1:500) antibodies followed, each for 1 h at room temperature. Fluorescein isothiocyanate (FITC) conjugated tyramide plus was then added for detection (1:750; Invitrogen, Carlsbad, California, USA). Next, the sections were microwaved to eliminate endogenous HRP activity and the second primary antibody (rabbit polyclonal anti-cyclinD1; Santa-Cruz sc-8396 (Santa Cruz Biotechnology, Inc. Heidelberg, Germany)) was used at a 1:500 dilution at 4°C overnight. The staining was then developed using ABC and biotinylated tyramide (1:5000) for 5 minutes at room temperature and streptavidin-594 (1:2000; Molecular Probes, Invitrogen, Carlsbad, California, USA) for 1 h at room temperature. The slides were mounted with 0.1 M Tris and viewed in a Leica DMR fluorescent microscope. The negative control omitting the primary antibody resulted in nostaining.

To determine the percentage of double-labeled mammary epithelial cells, an observer scored 500 to 1,000 cells for each combination of antibodies.

## Results

**Figure 1:**
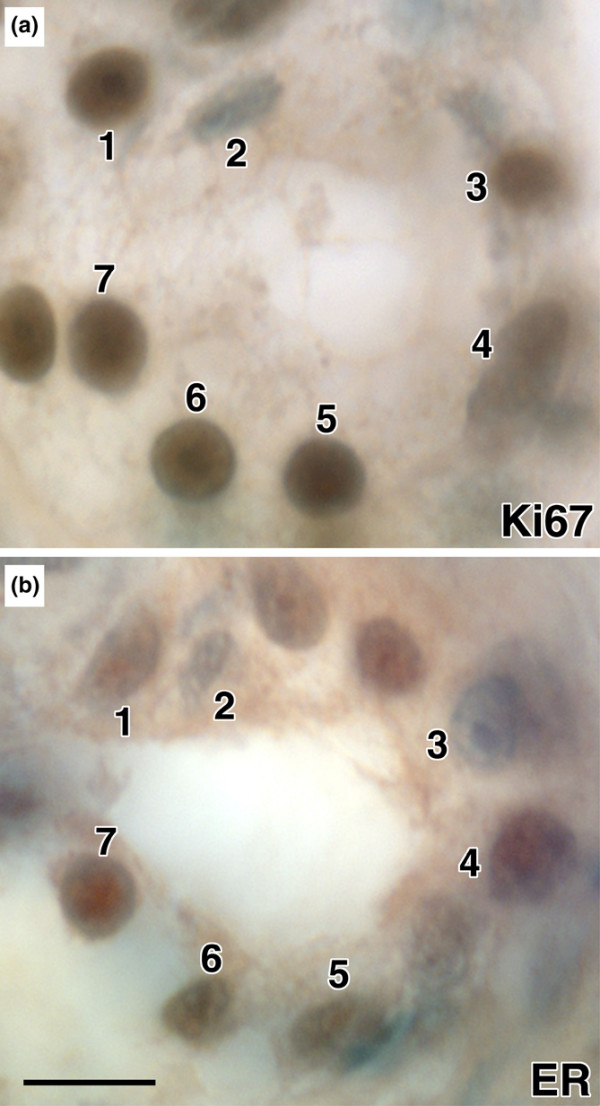
Co-localization of estrogen receptor (ER)α and Ki67 in primate mammary epithelial cells. **(a) **ERα and **(b) **Ki67 immunostaining (brown) are detected in the same epithelial cells on serial sections (3 μm) of primate mammary tissue, for example, cells 4–7. Bar = 20 μm.

To evaluate co-expression of ERα and the proliferation specific marker Ki67 we used immunohistochemical detection on thin (3 μm) serial sections through the mammary epithelium from estrogen-treated monkeys. We found that 23 ± 10% (mean ± standard deviation for the 4 estrogen treated animals; approximately 1,000 cells scored for each) of mammary epithelial cells were ER+/Ki67+ while 22 ± 2% were ER-/Ki67+ (Figure 1). Approximately 15% of cells were ERα+/Ki67-, and the remaining 40% was negative for both antigens.

**Figure 2:**
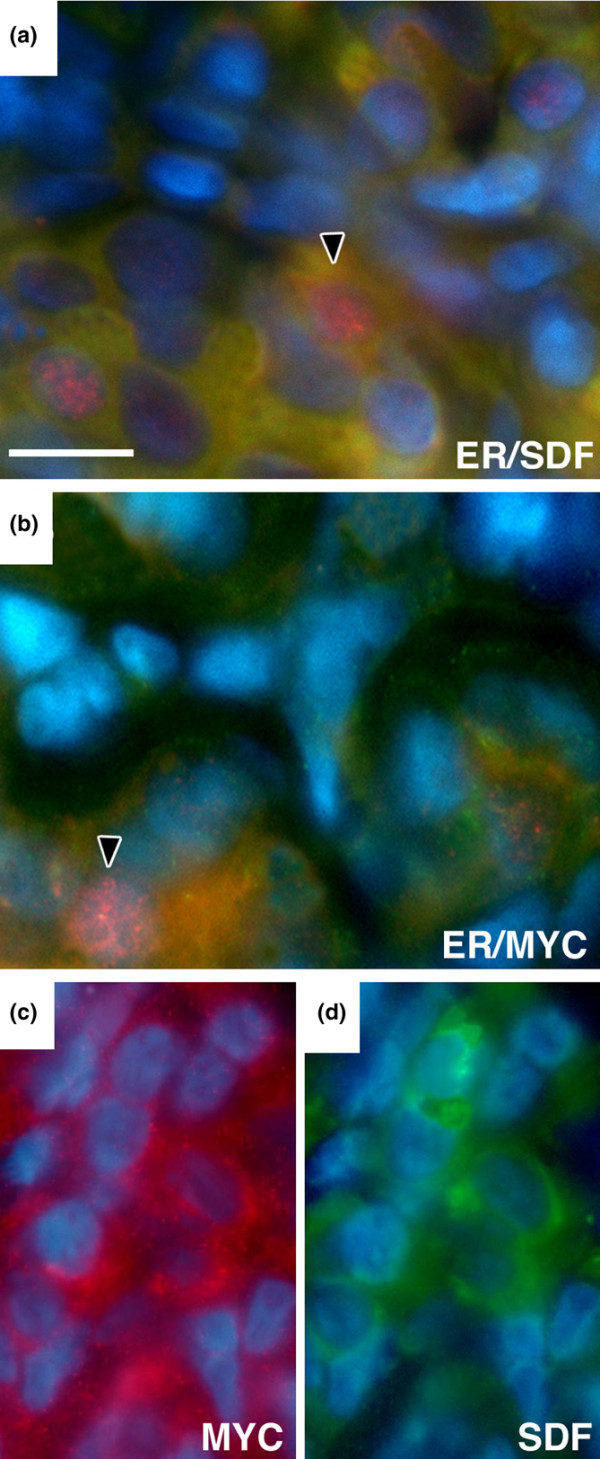
Co-expression of Erα and its molecular targets in mammary epithelium. (a) Erα is detected as a red nuclear stain and SDF-1 as a yellow cytoplasmic stain. (b) The red duclear stain for Erα is surrounded by yellow cytoplasmic signal from MYC immunostaining. Arrowheads in a and b point to cells where the Colocalization is clear. (c and d) serial sections show MYC (red) and SDF-1 (green) immunostaining in the same cells. Bar = 20 microns.

**Figure 3:**
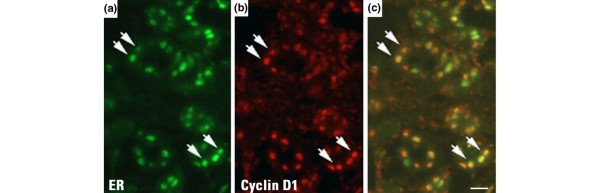
Colocalization of estrogen receptor (ER)α and cyclin D1 in mammary epithelial cells. Double immunofluorescence in the same tissue section shows: **(a) **ERα, green nuclear staining; **(b) **cyclinD1, red nuclear staining; **(c) **ERα and cyclinD1: yellow nuclear staining. Arrows indicate cells co-expressing ERα and cyclinD1. Bar = 20 μm.

To investigate the co-expression of the estrogen target molecules SDF-1, MYC and cyclin D1 in ERα positive cells, we used double immunofluorescence. Co-expression of ERα and SDF-1 was detected in 25% of cells while 9% and 19.3% of cells were stained positive for ERα alone and for SDF-1 alone, respectively (Figure 2, Table 1). The remaining 46.7% of cells was negative for both antigens. Approximately 26% of mammary epithelial cells stained positive for both ERα and MYC antibodies. No expression was observed in 48.6% of cells while 7.2% were ERα+/MYC- and 18% ERα-/MYC+. Most SDF-1 positive cells were also MYC positive, and vice versa (Figure 2c, d). All our data were consistent in four different estrogen treated animals. Finally, cyclin D1 was detected in over 90% of ERα positive cells (Figure 3).

## Discussion

This study has shown that ERα is co-expressed with the proliferation antigen Ki67 in a population of normal primate mammary epithelial cells under the conditions of short-term estrogen stimulation and high rates of proliferation. In addition, ERα is co-expressed with the estrogen-induced target proteins SDF-1, MYC and cyclin D1, which are all implicated in mammary epithelial cell proliferation. These observations indicate that it is likely that estrogen has direct mitogenic effects upon ERα-positive mammary epithelial cells and obviate the need to invoke unknown paracrine mediators of estrogen action in the mammary gland. Previous studies investigating ERα and Ki67 co-expression reported very low proliferation rates [4]. With only 1% to 2% of cells engaged in mitosis, if ERα and Ki67 co-expression occurs for 10% to 20% of the cell cycle, then even with 100% efficient histochemical methodology, only approximately 0.2% of cells would be both ERα and Ki67 positive, which is in the range of what has been reported. The high rate of mammary epithelial proliferation induced by estrogen treatment [8] in the present study provided a more optimal window of opportunity to detect transient co-expression of ERα and Ki67. The present results are certainly more compatible with estrogen's well-established ability to promote proliferation in ER-positive breast cancer cell lines [5].

MYC serves as a central component of estrogen regulation of cell cycle progression and oncogenesis [2]. ERα and MYC are frequently over-expressed during breast cancer progression [9]. We have previously shown that MYC expression is positively correlated with ERα expression in normal primate mammary epithelium [10]. In this study, using mammary tissue sections from the same animals, we show that more than one fourth of the cells co-express ERα and MYC, providing *in vivo *confirmation that MYC is implicated as a mediator of estrogenic induced proliferation. A novel and important finding of this study is that almost all the cells that express MYC also express SDF-1. SDF-1 has recently been implicated as a mediator of estrogen's proliferative actions in ovarian and breast cancer cells [3]. The present finding of co-localization of ERα and SDF-1 and MYC and SDF-1 provides the first confirmation that these interactions appear relevant in normal proliferating mammary tissue.

## Conclusion

ERα is expressed in proliferating mammary epithelial cells together with the estrogen-induced proteins MYC, cyclin D1 and SDF-1, consistent with a direct mitogenic action by estrogen in mammary epithelium.

## Abbreviations

ER = estrogen receptor; PBS = phosphate-buffered saline; SDF = stromal cell-derived factor.

## Competing interests

The authors declare that they have no competing interests.

## Authors' contributions

CD and JZ contributed to the conception and design of the study, performed immunohistochemistry experiments, statistics and interpretation of data. JW, JXW and DW performed immunohistochemistry experiments. EM, LM and JZ performed double immunofluorescence studies. CB contributed to the conception and design of the study and interpretation of data. All the authors read and approved the final manuscript.

## Figures and Tables

**Table 1:** Co-expression of estrogen receptor-α with MYC and stromal cell-derived factor-1 in mammary epithelial cells

Double stain	ERα (+) %	MYC (+) %	SDF (+) %	Both (+) %	None %
ERα/MYC	7.2	18		26.2	48.6
ERα/SDF	9		19.3	25	46.7

One thousand cells were counted for each combination of antibodies in mammary gland sections of four estrogen treated monkeys stained for estrogen receptor (ER)α/MYC and ERα/stromal cell-derived factor (SDF)-1 using double immunofluorescence.
